# Fading and Color Reproducibility of Nipple–Areola Tattoos in Asian Patients

**DOI:** 10.1055/a-2309-2731

**Published:** 2024-06-19

**Authors:** Mao Yamamoto, Hiroki Mori, Masako Akiyama, Mutsumi Okazaki

**Affiliations:** 1 Department of Plastic and Reconstructive Surgery, Graduate School of Medical and Dental Sciences, Tokyo Medical and Dental University, Tokyo, Japan; 2 Department of Plastic Surgery, Tsuchiura Kyodo General Hospital, Ibaraki, Japan; 3 Research Administration Divisions, Research University Promotion Organization, Tokyo Medical and Dental University, Tokyo, Japan; 4 Department of Plastic and Reconstructive Surgery, Graduate School of Medicine, The University of Tokyo, Tokyo, Japan

**Keywords:** nipple, tattoo, breast reconstruction, NAC

## Abstract

**Background** The purpose of this study was to clarify fading, red, green, and blue values (RGB) change, and color reproducibility for nipple–areola complex (NAC) tattoos.

**Methods** NAC tattooing was performed on 60 sites in 59 Japanese patients prospectively. The evaluation was assessed using digital photo, Casmatch standardization, and RGB and luminance values preoperatively, immediately after, 1 week, 1, 3, 6, and 12 months after tattooing. RGB and luminance values changes over time, time-adjusted fading rate, and the rate of luminance at 12 months were calculated. In color reproducibility study ( *n* = 34), RGB values after 12 months were compared with the color sample about dark/reddish and light/less reddish pigments.

**Results** RGB varied widely from immediately after to 1 month after tattooing. For RGB and luminance, significant differences were seen between pre and immediate after, 1 and 3 months, 3 and 6 months, and 6 and 12 months. In G values, significant differences were seen between all neighboring points. The fading rate tended to decrease as time progresses, but was not significant, that is, fading continued even between 6 and 12 months. Luminance was 9% brighter than contralateral NAC at 12 months. Color reproducibility tended to be higher with dark/reddish pigments, despite no significant differences.

**Conclusion** The fading rate of tattooed NACs tended to decrease as time progresses, but fading still occurs between 6 and 12 months. Luminance was 9% brighter than contralateral NAC at 12 months after.

## Introduction

Nipple–areola complex (NAC) tattoos were first reported in 1975 by Rees. 1 Many reports have now investigated tattoo techniques 2 3 4 5 6 and this method has been widely used to provide finishing touches in breast reconstruction. The choice of pigments is determined by discussions with the patient, mostly in comparison with the color on the healthy side. Even with careful consideration, tattoo colors gradually fade over time. 7 8 Many physicians perform tattooing empirically, and few reports have investigated its effects and continuity. 7 8 Most of those studies were retrospective investigations of Caucasian populations, and few prospective studies have examined tattooing. We therefore planned a prospective study of NAC tattoos in Asian patients.

The purpose of this study was to reveal: (1) how long does it take for the fading to stabilize, and what is the luminance rate of the affected side to the contralateral side in 1 year? and (2) which pigment is most reproducible for NAC tattoos compared with color samples?

## Methods

This study prospectively investigated patients who underwent NAC tattooing after breast reconstruction accompanied by cancer ablation between June 2010 and February 2014. This study was registered with the University hospital Medical Information Network (UMIN) clinical trials registry (UMIN000004986; https://center6.umin.ac.jp/cgi-openbin/ctr_e/ctr_view.cgi?recptno=R000005929 ).

During this period, NAC tattooing was performed on 60 sites in 59 Japanese patients, including one bilateral case reconstructed with abdominal skin ( Table 1 ). Duration of follow-up was defined as the period in months between the day of tattooing and the day of post-tattoo photo. NACs were tattooed using a tattooing machine and pigments (Permark; PMT Corporation, Chanhassen, MN) under local anesthesia. We chose “flesh” series, the kinds of color chart.

**Table 1:** Patient demographics

Patient, *n*	59 (60 sites)
Age (years)	51.3 (SD 9.3)
Breast reconstruction	
Abdominal flap	32
Latissimus dorsi flap	13
Breast implant	14
Breast conservation surgery with nipple resection	1
Nipple reconstruction	
Modified C–V flap	60
Pigments	
Flesh2.5	14
Flesh4	7
Flesh7	5
Flesh8	4
Flesh9	6
Flesh12	6
Others	18

Abbreviation: SD, standard deviation.

**Fig. 1:**
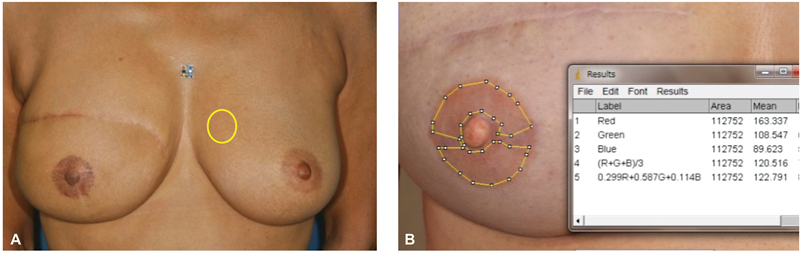
Method for measuring RGB values of NACs. ( **A** ) Yellow circle is normal breast skin near the nontattooed NAC. ( **B** ) Measurement site of the tattooed NAC. NAC, nipple–areola complex; RGB, red, green, and blue.

Photographs with a color reference marker (Casmatch; Bear Medic, Chiba, Japan) were taken preoperatively, immediately after tattooing, and 1 week, 1, 3, 6, and 12 months after tattooing. A single-lens digital camera (D90; NIKON, Tokyo, Japan) was used under the same fluorescent lighting conditions. Photographs were standardized in terms of luminance using Casmatch and Photoshop software (Adobe, San Jose, CA). Standardization was done according to the manufacturer's instructions: in Photoshop, the sample range of the eyedropper tool was set to an average of 5 pixel squares. In the Lighting Levels correction, enter 230 for white R, G, and B; 40 for black R, G, and B; 121 for gray R, G, and B, respectively. Open the photo with the Casmatch applied, select the white, black, and gray eyedropper, and click on the white, black, and gray Casmatch in the photo to correct the luminance. Then luminance and red, green, and blue (RGB) values for the tattooed NAC, contralateral NAC, and normal breast skin near the contralateral NAC ( Fig. 1A ) were analyzed using ImageJ software (U.S. National Institutes of Health, Bethesda, MD). We conducted calculations only for the areolar area of the NAC, because the nipple area was sometimes tattooed with a different color or affected by shadow. We also avoided taking measurements in areas containing operational scars because it is difficult to add pigment to scars ( Fig. 1B ).

### Study 1: Red, Green, and Blue, and Luminance Changes Over Time

**Fig. 2:**
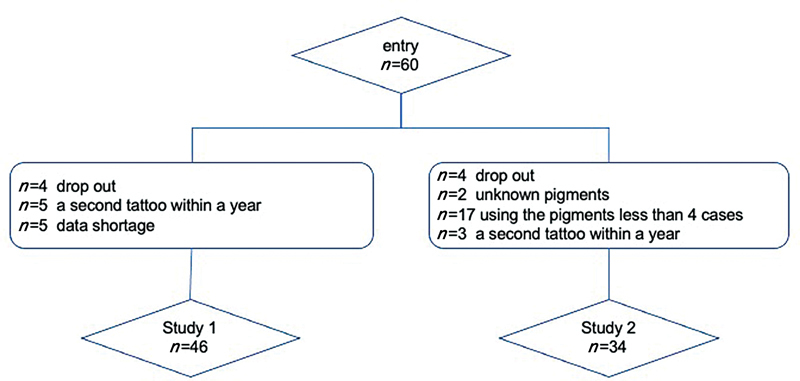
Flowchart showing patient selection.

This analysis was performed at 46 sites of 45 patients, after excluding 4 sites in 4 patients who dropped out, 5 sites in 5 patients who received a second tattoo within a year, and 4 sites in 4 patients for whom data were missing ( Fig. 2 ). The skin types used in NAC reconstruction were abdominal ( *n* = 25), dorsal ( *n* = 11), or breast skin ( *n* = 10). All nipple reconstruction methods are modified C–V flap.

This study further standardized RGB values by dividing RGB values for tattooed NAC by those for normal breast skin near the contralateral NAC. Accordingly, we compared the rate of RGB values for tattooed NAC with those for normal skin in this study.

RGB values were transformed to light intensity using a standard conversion. 9

Luminance = 0.298912R + 0.586611G + 0.114478B

Luminance is a photometric measure of the luminous intensity per unit area of light traveling in a given direction and represents the human perception of the brightness of the light source.

**Fig. 3:**
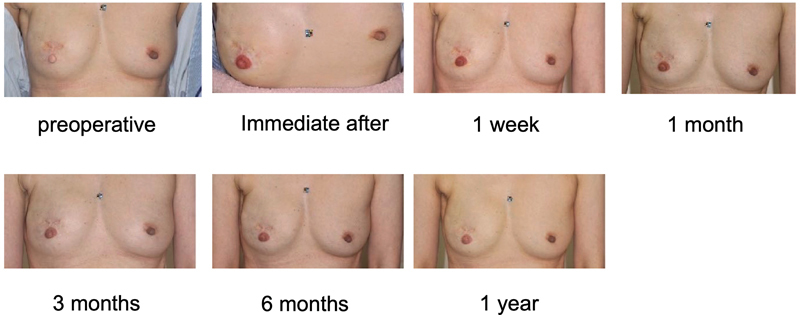
Representative progress of tattooed NAC. NAC, nipple-areola complex.

We evaluated the change in RGB and luminance between preoperatively, immediately after tattooing, and 1 week, 1, 3, 6, and 12 months after tattooing ( Fig. 3) .

To adjust the time frame, we also calculated the fading rate by dividing five periods, from immediately after tattooing to 1 week after, from 1 week to 1 month after, from 1 to 3 months after, from 3 to 6 months after, and from 6 months to 12 months after. The following formula was used to determine the fading rate:

Fading rate (per month) = (Luminance at later period− Luminance at earlier period)/Luminance at later period

Finally, we calculated the rate of tattooed/contralateral NAC luminance at 12 months for 44 cases (44 sides), excluding bilateral abdominal case. Furthermore, the rate of tattooed/contralateral NAC luminance was compared for the type of skin used for NAC reconstruction.

### Study 2: Reproducibility of Color

The analysis was performed for 34 sites in 33 patients after excluding 4 sites in 4 patients who dropped out, 2 sites in 2 patients with 2 unknown pigments, 17 sites in 17 patients with pigments used at less than 4 sites, and 3 sites in 3 patients (flesh2.5: 1, flesh4: 2) who received a second tattoo within a year ( Fig. 2 ). This analysis examined the pigments for flesh 2.5, 4, 7, 8, 9, and 12 (Permark; PMT Corporation, Chanhassen, MN), which were each used at four or more sites.

**Fig. 4:**
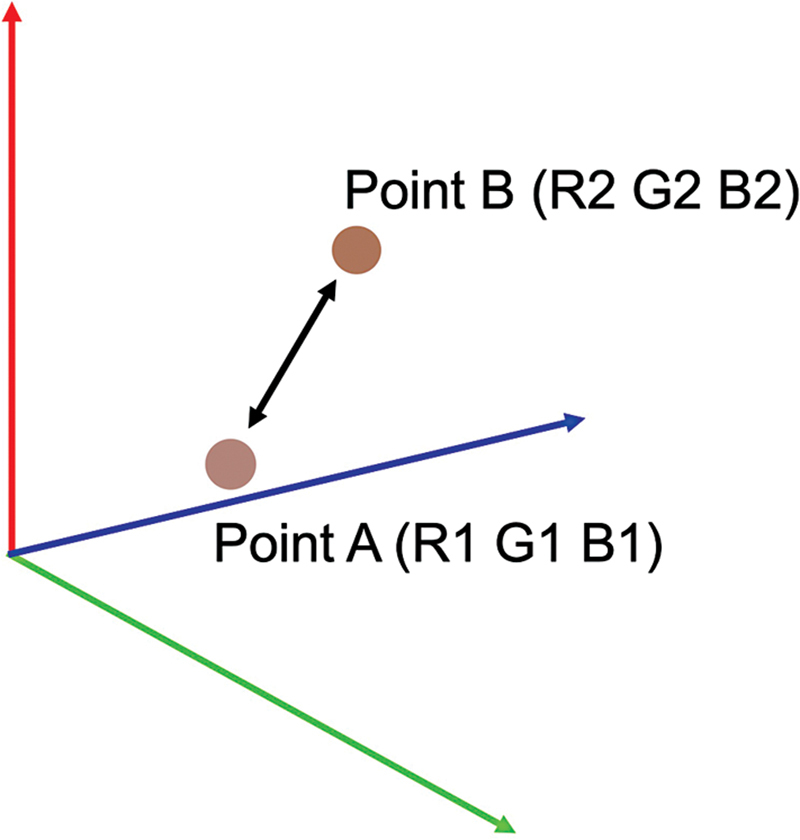
RGB distance between two points. A (R1, G1, B1), B (R2, G2, B2), the distance *D* . RGB, red, green, and blue. *D* = √([R2 − R1] ^2^ + [G2 − G1] ^2^ + [B2 − B1] ^2^ )

We also took photographs of the color samples of the tattoo pigments and standardized the images with Casmatch. We regarded RGB values as three-dimensional data and calculated the distance between the two points. That is, given the two points A (R1, G1, B1) and B (R2, G2, B2), the distance ( *D* ) between these points was calculated using the formula:

*D* = √([R2 − R1]2 + [G2 − G1]2 + [B2 − B1]2) ( Fig. 4 )

Regarding color reproducibility, we targeted RGB values after 12 months. We compared the RGB values of the tattooed part with those of the color samples.

### Statistics

All statistical analyses were performed using EZR, which is a modified version of R Commander designed to add statistical functions frequently used in biostatistics. 10 In changes over time study, repeated-measures analysis of variance (ANOVA) was performed to assess whether there was an overall difference between time points. We then compared the differences at each time point using paired *t* -test and Bonferroni correction while one-way ANOVA or unpaired *t* -test was used in other study. All tests with *p* -value less than 0.05 were considered significant.

## Results

### Study 1

**Fig. 5:**
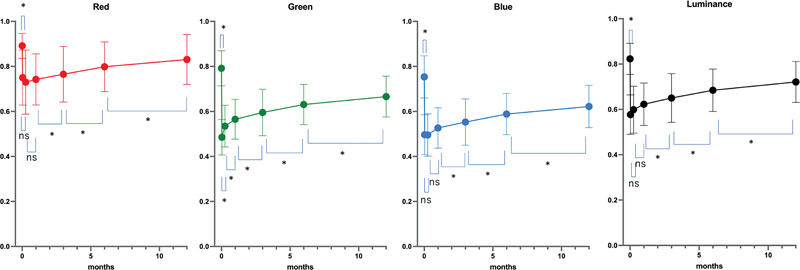
RGB and luminance change in tattooed NACs ( *n* = 46). Rate of RGB values for tattooed NAC with those for normal skin is shown. Plots and bars show mean and standard deviation (SD) trends. Asterisks indicate significant differences in repeated-measures ANOVA. ANOVA, NAC, nipple–areola complex; RGB, red, green, and blue; ns, not significant.

R and B values were smallest at 1 week after tattooing, and G and luminance values were smallest at immediate after tattooing. We used repeated-measures ANOVA to confirm terms in which the RGB and luminance values changed significantly (R: <0.0001, G: <0.0001, B: <0.0001, Luminance: <0.0001), and in which significant differences existed between measurement time points using paired *t* -test and Bonferroni correction. In all elements, significant differences were seen between pre and immediately after, 1 and 3 months, 3 and 6 months, and 6 and 12 months. In case of G values, significant differences were observed between all neighboring points ( Fig. 5 ; Supplementary Table S1 [available in the online version only]).

**Fig. 6:**
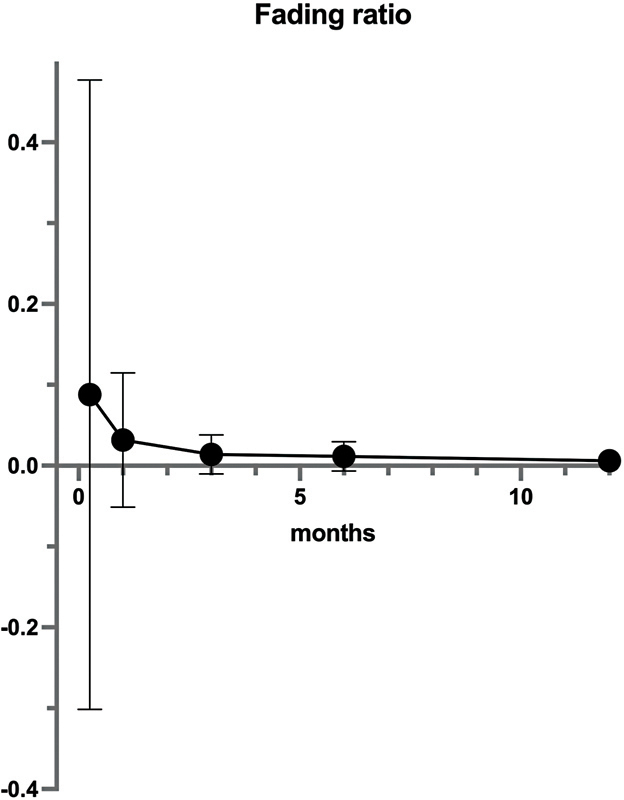
Fading rate from immediately after to 12 months after tattooing. Plots and bars show mean and SD trends. No significant differences were identified between the five terms ( *p* = 0.19) in repeated-measures ANOVA. ANOVA, SD, standard deviation.

The fading rate tended to decrease gradually from immediately after to 1 week, from 1 week to 1 month, from 1 to 3 months, from 3 to 6 months, and from 6 to 12 months, and the standard deviation also tended to decrease. We also used repeated-measures ANOVA for comparisons between fading rates in the five terms, but no significant differences were identified ( *p* = 0.19; Fig. 6 , Supplementary Table S2 [available in the online version only]).

**Fig. 7:**
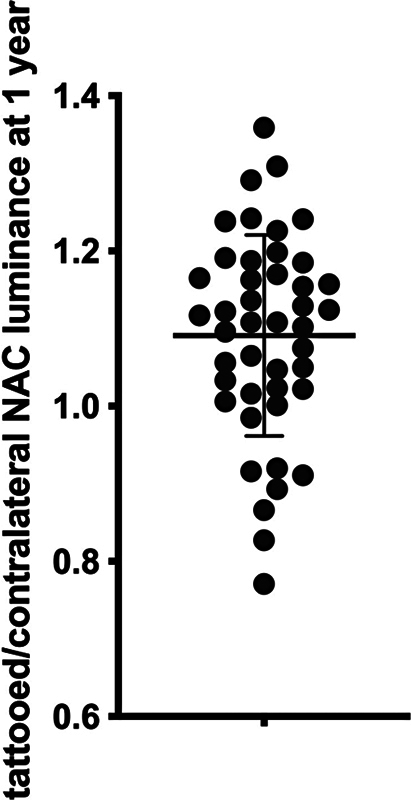
Rate of tattooed/contralateral NAC luminance at 12 months. Bars show mean and SD trends. NAC, nipple–areola complex; SD, standard deviation.

At 12 months after tattooing, the rate of tattooed/contralateral NAC luminance was mean 1.09 ± 0.13 ( Fig. 7 ). No significant differences were observed in one-way ANOVA between the rates of the three parts of skin from different donor sites ( *p* = 0.63), with 1.11 ± 0.13 for the abdomen skin ( *n* = 23), 1.08 ± 0.12 for dorsal skin ( *n* = 11), and 1.06 ± 0.16 for the breast skin ( *n* = 10).

### Study 2

Subjects showed flesh2.5 ( *n* = 12), flesh4 ( *n* = 4), flesh7 ( *n* = 4), flesh8 ( *n* = 4), flesh9 ( *n* = 5), and flesh12 sites ( *n* = 5). We tattooed one patient with flesh7 at both NACs.

**Fig. 8:**
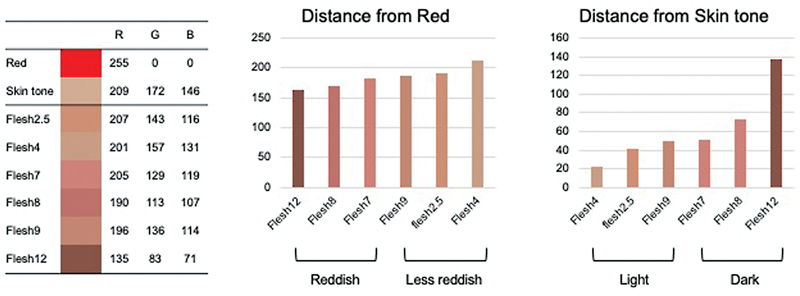
Classification of pigments by distance to Red or skin tone. The left table shows the RGB elements of each pigment. The center graph shows the distance from red in each pigment. The right graph shows the distance from skin tone in each pigment. From these, we defined flesh7, 8, and 12 as “reddish/dark,” and flesh2.5, 4, and 9 as “less reddish/light.” RGB, red, green, and blue.

**Fig. 9:**
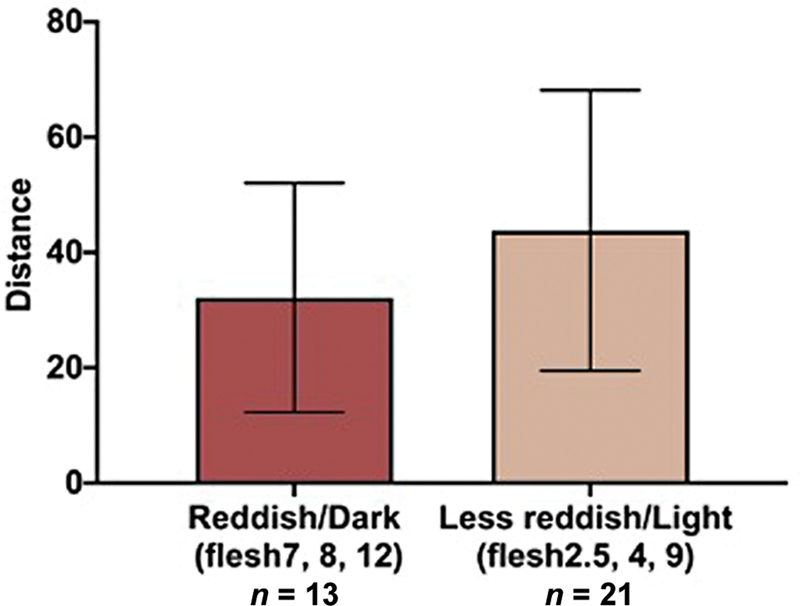
Distances between tattooed skin and color sample of the pigment in the RGB dimension. The tattooed skin by reddish/dark (flesh7, 8, and 12) pigment tended to be closer to the color sample than one by less reddish/light (flesh2.5, 4, and 9) pigment in unpaired *t* -test, but no significant differences were identified ( *p* = 0.16). Boxes and bars show mean and SD trends. RGB, red, green, and blue; SD, standard deviation.

RGB value of each pigment and the distance from Red (R 255, G 0, B 0) and skin tone to each pigment are shown in Fig. 8 . Skin tone is defined as the mean value (R 209, G 172, B 146) of normal breast skin near the contralateral NAC preoperatively. Thereby, flesh7, 8, and 12 were defined as “reddish/dark,” and flesh2.5, 4, and 9 as “less reddish/light.” In both groups, distances between tattooed skin and color sample of the pigment in the RGB dimension were evaluated. The “reddish/dark” tended to be closer to the color sample than “less reddish/light” in unpaired *t* -test, but no significant differences were identified ( *p* = 0.16; Fig. 9 ).

## Discussion

In this study, we showed that (1) RGB and luminance significantly changed and faded from 1 to 3, 3 to 6, and 6 to 12 months postoperatively, and (2) time-adjusted fading rates showed a trend of decreasing fading during the same period, but no significant difference, that is, fading continued even between 6 and 12 months, and (3) luminance was 9% brighter than contralateral NAC at 12 months after, and (4) color reproducibility tended to be higher with dark or reddish pigments.

During the early stages of acute inflammatory reaction after tattooing, keratinocytes take up pigment particles and cells such as macrophages infiltrate the epidermis via the destroyed basement membrane. 8 In the present results, despite the smallest luminance and G value immediately after, the R and B values were the smallest 1 week later. And the fading rate was shown to vary widely from immediately after to 1 week. These are presumed to be due to unstable color, with bleeding, hemorrhagic plaques, inflammation, and crust formation occurring immediately and for several weeks thereafter.

After a month, removal of pigment particles through the epidermis continues, but once the basement membrane has reformed at the epidermal–dermal junction, the concentration of pigment particles in the epidermis decreases. The epidermal–dermal junction normalizes, and this process stops. Pigment particles are found only in dermal fibroblasts. 8 For this reason, the tattoo color is expected to change to some extent. Spear and Arias reported that 9.5% of tattoos required touch-up during his study, 11 and Eskenazi noted that 10% of patients requested additional tattooing. 12 Furthermore, Hugo et al noted that 40% of tattooed NACs were blighter in color than the color of tattooed NACs during the postoperative period and El-Ali et al noted fading of the tattooed NAC in 34% of cases. 7 13 Even if the skin structure returns to normal, external stimuli such as ultraviolet radiation and aging may contribute to fading. 14 The present results support these reports, and some fading of tattooed NACs is inevitable. On the other hand, the fading rate between 6 and 12 months after tattooing is quite small, although not significantly different, suggesting that the change in luminance after 6 months is mild.

Comparing luminance of the tattooed and contralateral NACs for 12 months after tattooing, the tattooed side basically was brighter than the contralateral side. El-Ali et al also noted that tattoo color faded and symmetry between normal and tattooed sides was 0.91 in color intensity values. 7 The luminance rate between tattooed and contralateral sides in our study was 1.09, showing similar results to those described by El-Ali et al.

In terms of color reproducibility, pigments closer to red and darker than skin color tended to reproduce better, while colors farther from red and closer to skin tended to reproduce less well. One reason was that bleeding made it difficult to judge whether sufficient tattooing had been achieved, resulting in insufficient tattooing. In fact, “less reddish/light” pigment (flesh2.5, 1 and flesh4, 2) patients underwent a second tattooing. This did not occur with “reddish/dark” pigment. El-Ali et al recommended that tattoos start at about one-third darker than the color of the nipple on the normal side, 7 but since a few cases in the current study occurred darker than the healthy side, our current goal is the same color as the color chart, and a second tattoo is performed if it is blighter.

The clinical significance of this study is that it shows objective data on tattoo fading in Asians, along with RGB changes, that the fading rate tends to decrease over time, but still fades between 6 and 12 months, and that the color reproducibility may vary depending on the inked color. These findings are useful to explain to patients.

Several limitations to this study must be kept in mind. As these data used values of luminance with RGB values, we did not evaluate differences in color accurately. There are various ways to represent color, including RGB, CMYK, and Laboratory, and there is some controversy as to whether RGB is appropriate for color evaluation. 15 However, to evaluate digital photographs on a monitor, we used RGB, which is the color representation method in monitors. Additionally, the sample size for each pigment was small in Study 2. Despite these limitations, this study represents the first prospective evaluation of luminance, fading rate, and color reproducibility for tattooed NACs in Asian patients.

Although no Food and Drug Administration-approved pigments for medical tattooing have been released and fading is inevitable, tattoos containing iron oxide and titanium dioxide can be safely provided. 16 When the color of a tattoo becomes stable is still unclear, so follow-up is warranted where possible.

### Conclusion

Prospective evaluation of RGB, luminance, fading rate, and color reproducibility for tattooed NACs was performed in Asian patients. RGB and luminance varied widely from immediately after to 1 month after tattooing. The fading rate tended to decrease as time progresses but fading is significant and still occurs between 6 and 12 months. Luminance was 9% brighter than contralateral NAC at 12 months after. Color reproducibility tended to be higher with reddish/dark pigments.
