# Supplementary material for: Fading and Color Reproducibility of Nipple–Areola Tattoos in Asian Patients
Source: Arch Plast Surg. 2024 Jun 19;51(4):356–62. doi: 10.1055/a-2309-2731 (PMC11288766; doi:10.1055/a-2309-2731)
Supplement: Supplementary file 1 — Supplementary Material [file 10-1055-a-2309-2731-s23aug0438oa.pdf]

Supplementary Table S1 Supplementary data for Fig. 5

	Red adj *p*	Green adj *p*	Blue adj *p*	Luminance adj *p*
Pre- to immediately after	<0.001	<0.001	<0.001	<0.001
Immediately after to 1 week	0.93	0.013	1	0.80
1 week to 1 month	1	0.012	0.094	0.079
1 to 3 months	0.022	0.0011	0.036	0.0021
3 to 6 months	0.0018	0.0011	0.0095	<0.001
6 to 12 months	0.0036	<0.001	0.025	<0.001

Abbreviation: adj: adjusted.

Supplementary Table S2 Supplementary data for Fig. 6

	Mean	SD
Immediately after to 1 week	0.091	0.39
1 week to 1 month	0.033	0.083
1 to 3 months	0.014	0.024
3 to 6 months	0.012	0.018
6 to 12 months	0.0060	0.0089

Abbreviation: SD, standard deviation.

 © 2024. The Author(s).
